# Assessment of compliance to the guidelines on nutritional support in a neonatal intensive care unit: association with an electronic health Records implementation

**DOI:** 10.1186/s12911-026-03533-x

**Published:** 2026-05-14

**Authors:** Camille Grelon, Mathilde Yverneau, Mathilde Prodhomme, Arnaud Coursin, Boris Delange, Alain Beuchée

**Affiliations:** 1 CHU Rennes, Rennes, France; 2 University of Rennes, Inserm, LTSI-UMR 1099, Rennes, F-35000 France

**Keywords:** Parenteral nutrition, Preterm infants, Electronic medical records, Postnatal growth

## Abstract

**Aim:**

The aim of the study was to measure compliance with parenteral nutrition guidelines after the implementation of individualized prescription software for extremely low-birth-weight preterm infants and the impact of these guidelines on postnatal growth at 36 weeks.

**Materials and methods:**

All newborns with a birthweight of less than 1000 g who were born between January 2012 and December 2015 (period A), between January 2019 and September 2021 (period B), without malformation or congenital anomalies, and admitted to the University Hospital of Rennes within the first 24 hours of life were included. Compliance to the ESPGHAN recommendations was compared between Periods A and B. Caloric and protein intakes were considered compliant if both the median caloric and protein intakes over days 5 to 7 were in agreement with the 2018 ESPGHAN recommendations. The association between the weight delta Z-score and weight at birth and at 36 weeks was assessed by multivariate analysis after weighting for the propensity score for adequate early intake.

**Results:**

Following the implementation of individualized prescription software, compliance to ESPGHAN recommendations significantly increased. In period B, there was 82% compliance to the protein intake recommendations from D5 to D7 and 63% agreement for total calories. After adjustment and weighting, a correlation was observed between noncompliance intake and reduced growth velocity, measured as the weight delta Z-score of weight. However, this correlation was not significantly associated with a lower rate of EUGR.

**Conclusion:**

The deployment of individualized prescription software with responsive calculations of anticipated intakes was associated with an improvement in the compliance of parenteral nutrition guidelines for preterm infants.

**Supplementary Information:**

The online version contains supplementary material available at 10.1186/s12911-026-03533-x.

## Introduction

Optimal nutrition for preterm infants is a major challenge in neonatal care because this is crucial to good postnatal growth and improves the neurological outcome. Premature infants with extremely low birth weights are at greater risk of extrauterine growth restriction (EUGR), which is associated with inadequate growth during hospitalization [1]. Optimizing weight gain is associated with better cognitive outcomes at 24 months in this population of premature newborns at greater risk of neurodevelopmental disorders [2, 3]. On the other hand, EUGR is associated with a poorer neurological prognosis [4]. Optimized nutritional management of protein and energy intake reduces the risk of EUGR and improves cognitive outcomes at 18 months [5, 6]. This management includes parenteral nutrition after birth due to digestive immaturity, which requires a very gradual increase in enteral feeding.

The amount of energy and protein required for optimal growth has yet to be determined. In 2005, ESPGHAN stated on the amount of energy and protein required for optimal growth in preterm infants, with an objective of 4–4.5 g/kg/day for protein intake [7]. These recommendations were then revised in 2018 with the aim of not exceeding 3.5 g of protein/kg/day due to the low level of evidence that led to the previous recommendations, and increasing caloric intake particularly lipid intake [8–11]. The two protocols are presented in Table 1. The latest recommendations also stated that a computerized prescription should be used in the ordering process of PN when possible [12].

**Table 1:** Local protocols for parenteral nutrition during periods A and B

	Period A	Period B
**Hydric intake (ml/kg/d)**		
Initial dose	80 - 100	80 - 100
Target dose	140–160	140–160
**Protein (g/kg/d)**		
Initial dose	2.4	1.5–2.5
Target dose	4–4.5	2.5–3.5
**Carbohydrates (g/kg/d)**		
Initial dose	6	5.8
Target dose	14–16	11.5–14.4 (min 5.8; max 17.3)
**Lipid (g/kg/d)**		
Initial dose	1	1–1.5
Target dose	3.5	4
**Calories (kcal/kg/d)**		
Initial dose	80	45–55
Target dose	110 - 120	90 - 120 (max 135) 65 of carbohydrate-lipid intake

The daily application of these new recommendations, as close as possible to patients’ needs, has been facilitated by the introduction of prescription software (Metavision - iMD-Soft) with a specific tab displaying past and planned calculated nutrient intakes, as well as recommended targets according to birth weight, gestational age at birth and postnatal age. The MetaVision ICU clinical information system is a patient data management solution designed to support clinical workflows and meet the requirements of adult, neonatal, and pediatric intensive care units. It continuously records electronic medical data in real time, providing clinicians with a unified patient flowsheet that consolidates relevant information. This integration of patient data supports clinical decision-making and individualized interventions, particularly for monitoring nutritional intake, electrolyte balance, fluid management, and weight progression. It dynamically adjusts the visualization of anticipated intakes based on the trial-and-error process of daily prescription adjustments. The software has been specifically configured to track intake and nutritional interventions in premature neonates, thus enhancing the visualization of information regarding recommendations, past intakes, anticipated intakes within the next 24 hours, and the infant’s growth. In clinical practice, metabolic complications and fluid restrictions can be important obstacles for adherence to guidelines [13]. A 2009 survey showed that the nutritional recommendations were poorly followed, with 57% of centers achieving the recommended energy intake [14]. Compliance to nutritional recommendations has been proposed as a possible solution for improving growth and reducing EUGR [15]

This study aimed to measure compliance at day 5 to 7 to international guidelines for parenteral nutrition in extremely low-birth-weight preterm infants before and after the implementation of an electronic health record. The secondary outcomes were to assess compliance to the guidelines on the first day and to assess postnatal growth with intakes in line with the recommendations.

## Materials and methods

### Study design and patient population

A retrospective monocentric observational study was conducted at level 3 NICU of the University Hospital of Rennes. The data were collected from January 2012 to December 2015 (period A) and from January 2019 to September 2021 (period B). All children born with a GA < 32 weeks and birth weight (BW) <1000 g during period B without malformations or congenital genetic abnormalities and who were hospitalized at the University Hospital of Rennes within the first 24 hours of life were included. This cohort was compared with a retrospective historical cohort comprising data from January 2012 to December 2015, which corresponds to period A [16]. Although the original database for period A included neonates with a birth weight < 1500 g, a strictly comparable subgroup was selected for the present analysis. Specifically, only infants meeting the same inclusion criteria as those in period B (GA < 32 weeks and BW < 1000 g) were retained in both cohorts. The lower inclusion limits for both cohorts were 24 weeks of GA and 500 g in birth weight. Newborns hospitalized for less than 7 days, whether due to death or discharge, at the University Hospital of Rennes were excluded. The sole inclusion criterion was the administration of PN during at least the first 7 consecutive days of life. The Rennes University Hospital Ethics Committee validated the study. The data were collected retrospectively in anonymized files from medical records.

### Nutrition protocol

The PN protocols analyzed in this study were based on the ESPGHAN recommendations [7–11]. The protocols applied during periods A and B used formulations composed of various PN solutions, which are listed in Table 1. The formulations used contained glucose, amino acids and lipids: Clinoleic® or SMOF lipid® for lipids, SNPN for protein intake mainly, and Pediaven® NN1 or 2 in the absence of a central line (Appendix 1). In both periods, standardized parenteral nutrition was preferred over individualized parenteral nutrition [17], and the recommended vascular approach for parenteral nutrition was the central route. Parenteral nutrition was initiated on Day 1 for all patients. The protocol relied on standardized binary protein-glucose solutions (locally produced SNPN, PEDIAVEN-1, PEDIAVEN-2, or two-compartment NUMETAH G13) administered in parallel with a lipid emulsion (SMOFLIPID or CLINOLEIC). To meet individual requirements from Day 1 to Day 7, these solutions were supplemented with glucose (at concentrations of 2.5%, 5%, 10%, or 30%) and electrolytes via Y-access. This daily titration allowed for precise adjustment of hydration and electrolytes, while optimizing the glucose-to-protein ratio according to the patient’s clinical status.

As Métavision was implemented at the University Hospital of Rennes in October 2018, the entire B population was able to benefit from this prescription model. A specific configuration of the Métavision® computerized patient file (iMDsoft®) integrating the reactive visualization of macro- and micronutrient intakes, past and anticipated according to the planned prescription, recommended objectives according to the context (GA and BW), and postnatal length and weight growth on reference curves facilitates daily application and is as close as possible to the parenteral nutrition protocol (Appendix 2) [18].

### Variables and data management

The primary outcome of this study was compliance with ESPGHAN recommendations regarding macronutrients and energy intake at day 5 to 7 of PN. This was assessed by examining the fraction of patients whose nutrient and energy provision was in the range of the recommended intake. Secondary outcomes were compliance at day 1; the delta Z-score for weight between birth and 36 weeks postmenstrual age and EUGR, defined as a weight Z-score decline greater than 1 (delta Z-score < −1) between birth and 36 weeks of postmenstrual age, as defined by Peila et al. [19]. The analyzed obstetric and neonatal demographic data of the patients are listed in Table 2.

**Table 2:** Baseline characteristics of the population during periods A and B

	N	Period A, *N* = 121	Period B, *N* = 97	*p* value
Choriomniotitis	217			
Yes		22 (18.3%)	34 (35.1%)	0.006
Antenatal corticosteroid	218			
Yes		110 (90.9%)	77 (79.4%)	0.018
Delivery	218			
Ceasarien		84 (69.4%)	59 (60.8%)	0.189
Multiparity	218			
Yes		34 (28.1%)	56 (57.7%)	<0.001
Preeclampsia	218			
Yes		38 (31.4%)	31 (32%)	0.93
PROM > 24 hours	218			
Yes		26 (21.5%)	18 (18.6%)	0.6
Apgar 1’	218			0.001
<5		41 (33.9%)	53 (54.6%)	
≥5		75 (62%)	44 (45.4%)	
Missing information		5 (4.13%)	0	
Apgar 5’	218			0.2
<5		14 (11.6%)	14 (14.4%)	
≥5		103 (85.1%)	83 (85.6%)	
Missing information		4 (3.31%)	0	
Apgar 10’	218			<0.001
<7		7 (5.79%)	13 (13.4%)	
≥7		79 (65.3%)	82 (84.5%)	
Missing information		35 (28.9%)	2 (2.06%)	
Intubation	218			
Yes		88 (72.7%)	69 (71.1%)	0.795
Surfactant	218			
Yes		110 (90.9%)	70 (72.2%)	0.001
Gestational age (weeks), median (IIQ)	218	26.90 (26.10, 28.00)	26.60 (25.70, 28.00)	0.292
Gender	218			0.3
Girl		64 (52.9%)	59 (60.8%)	
Boy		57 (47.1%)	38 (39.2%)	
Birth weight, median (IIQ)	218	860 (750, 930)	795 (690, 875)	0.008
Head circumference at birth, median (IIQ)	198	24.00 (23.00, 24.00)	23.00 (22.4, 24.5)	0.305
Birth length, median (IIQ)	194	34.0 [32.0;35.0]	33.0 [32.0;35.0]	0.089

BW: birth weight; GA: gestational age; HC: head circumference; BL: birth length; PROM: prolonged rupture of membranes

#### Anthropometric data

Anthropometric data at birth and during hospitalization, such as weight, length, and head circumference (HC), were retrospectively extracted from the University Hospital of Rennes data warehouse (eHOP) using the Shiny web application for data visualization and analysis. This interactive Shiny web application allows for initial quality control and extraction of data from Métavision®. It allows importing and anonymizing data from Métavision® and then visualizing, manipulating, validating, cleaning, and exporting them for further analysis. Any outliers or missing values were manually checked in the patient’s source record using an association table between pseudonyms and permanent patient identifiers.

#### Nutritional data

Daily nutritional intakes were also extracted from calculations made using Métavision® software (iMDsoft®) and a Shiny web application for data display and analysis. The Metavision® software calculates the prescribed intake as well as the intake truly received by the patient. Nutritional intake includes enteral and parenteral nutrition, as well as intake associated with medication. Nutritional data collected on the first day of life and from days 5 to 7 were studied. The initial dose refers to the actual intake received by the neonate at 4pm of the first complete finalized day (Day 1). This corresponds to the cumulative intake from 4pm to 4pm on the day that includes H24. While the exact postnatal age at measurement may vary slightly among infants, the central tendency remains Day 1. The collected data represents the actual intake received, rather than the prescribed dose.

#### Comorbidities

The following comorbidities during the hospital stay were recorded: grade 2 or higher bronchopulmonary dysplasia (BPD) according to the revised NICHD definition [20], patent ductus arteriosus (PDA), grade 2a or higher necrotizing enterocolitis (NEC) according to the modified Bell’s staging criteria [21], bacterial infection, retinopathy of prematurity (ROP), and neuroimaging lesions. The patients’ data were recorded based on medical discharge reports or medical files. “Severe comorbidities” were defined as stage 4 retinopathy, bronchopulmonary dysplasia and neuroimaging lesions such as lesions of the white matter or basal ganglia.

#### Postnatal growth assessment

Newborn weight was measured at birth and then daily or once every other day. Length and head circumference were measured once a week from the first week of life, with a length board and a paper tape, respectively. All anthropometric data, including birth weight and weight at 36 weeks postmenstrual age, were converted into Z-scores using Fenton curves [22].

### Statistical analysis

Categorical variables are summarized as counts and relative amounts (%). Continuous variables were tested for normality (Q‒Q plots and Shapiro test) and are presented as medians [25p, 75p].

Given the retrospective nature of our study and the fixed sample size, a post-hoc power analysis was conducted to assess whether the study had sufficient power to detect meaningful differences in compliance rates. We estimated the Minimal Detectable Effect (MDE) at a 5% significance level and 80% power, using standard methods for two-proportion comparisons. The MDE represents the smallest difference that could be reliably detected with the given sample size. The specific results of this analysis are reported in the Results section.

The caloric and protein intakes were considered compliant if, for each patient, both the median caloric and protein intakes on days 5 to 7 were in line with the ESPGHAN recommendations [7–11]. Any deviation, whether lower or excessive intake, was classified as “noncompliance.”

To analyze the effect of noncompliance caloric and/or protein intake on postnatal growth, confounding factors that may affect the nutritional intervention modality were considered by estimating the propensity score. A propensity score was calculated for each patient from both periods through a logistic regression model, with adequacy of caloric and/or protein intake as the dependent variable and the following characteristics as covariates: gestational age, sex, birth weight Z-score, antenatal corticosteroid therapy, prolonged rupture of membranes (>24 hours), preeclampsia, multiparity, chorioamnionitis, mode of delivery, 1- and 5-minute APGAR scores, intubation, and surfactant administration. The quality of the weighting based on propensity scores was assessed by estimating standardized mean differences (SMDs). The “matching weight” (MW) method was found to be the most effective method, with the lowest residual imbalance (Appendix 3) [23]. Patients from both periods with noncompliance caloric and/or protein intakes were then compared to those with compliant caloric and protein intakes on days 5 to 7 after weighting by the MW transform of the propensity score.

Odds ratios were estimated to quantify the association between noncompliance caloric and/or protein intake and EUGR using both unweighted and MW-weighted generalized linear modeling with binomial family and logit link function. The best model was selected by stepwise minimization of the Akaike information criterion (AIC). Additionally, the association between noncompliance caloric and/or protein intake and postnatal growth was assessed using both unweighted and MW-weighted generalized linear model with a Gaussian distribution and an identity link function, where postnatal growth was defined as the difference in Z-scores between birth weight and weight at 36 weeks postmenstrual age, treated as a continuous variable.

The significance level was set at *p* < 0.05. All the data were analyzed with R® (R Core Team (2021)). R: A language and environment for statistical computing. R Foundation for Statistical Computing, Vienna, Austria. URL)

## Results

### Patient demographics

**Fig. 1:**
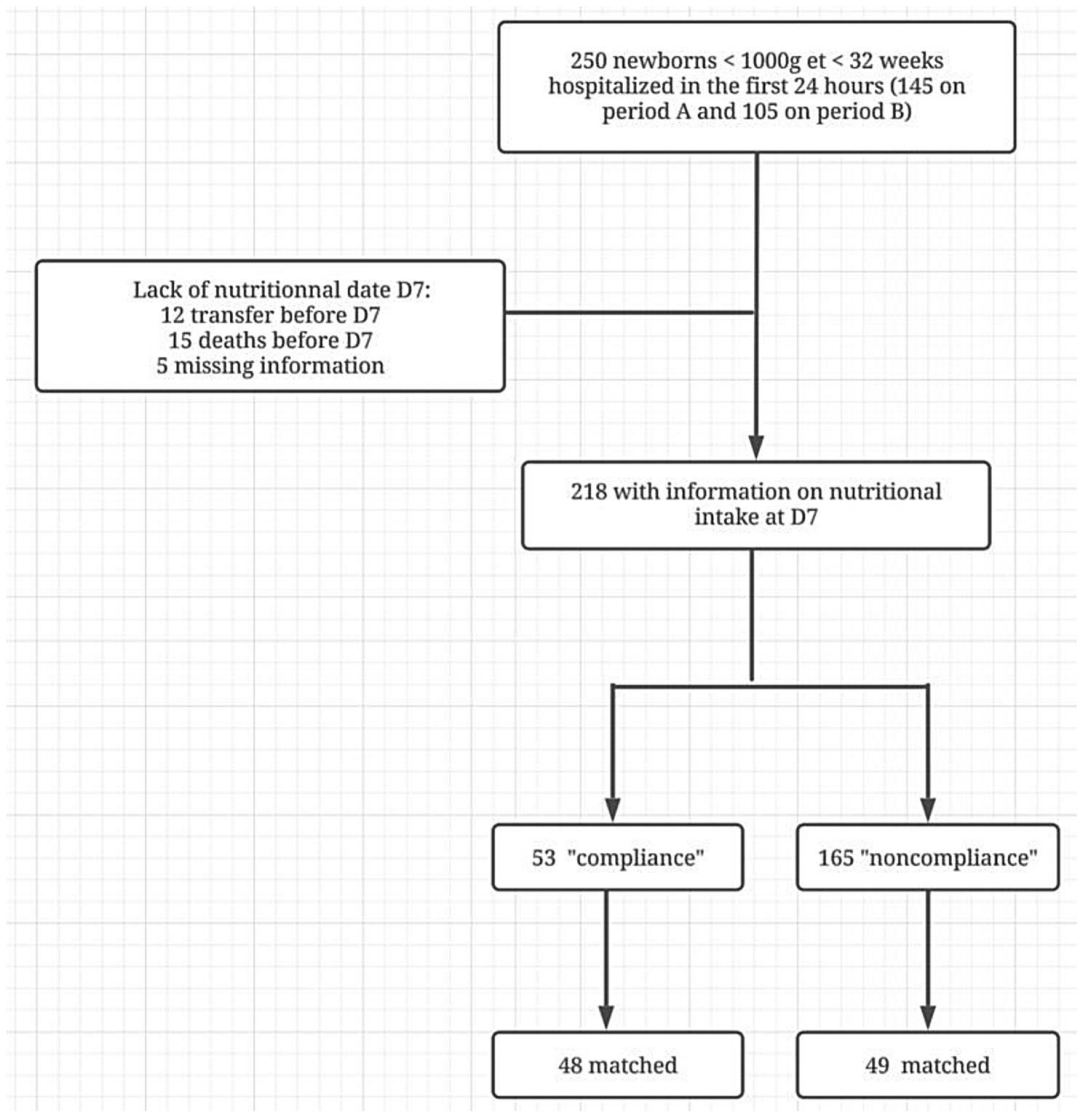
Flow chart

A total of 250 neonates were identified for inclusion. Twelve were excluded due to a length of stay of less than 7 days, fifteen died before D7, and nutritional data at D7 were missing for five. After these exclusions, 218 neonates were included in the analysis (121 in period A and 97 in period B). Among the study population (*n* = 218), 31 infants died before reaching 36 weeks of PMA, and a total of 34 died before hospital discharge (Fig. 1).

Table 2 summarizes the patient obstetrical and neonatal characteristics during the two periods. The evolution of GA at birth and BW over periods A and B are shown in Table 2. GA and the Z score of BW were comparable between periods A and B (26.9 SA [26.1;28.0] vs 26.6 SA [25.7;28.0] and −0.38 [−1.15,0.1] vs. −0.58 [−1.22;0.09], respectively).

### Nutritional intake and compliance with the guidelines

**Fig. 2:**
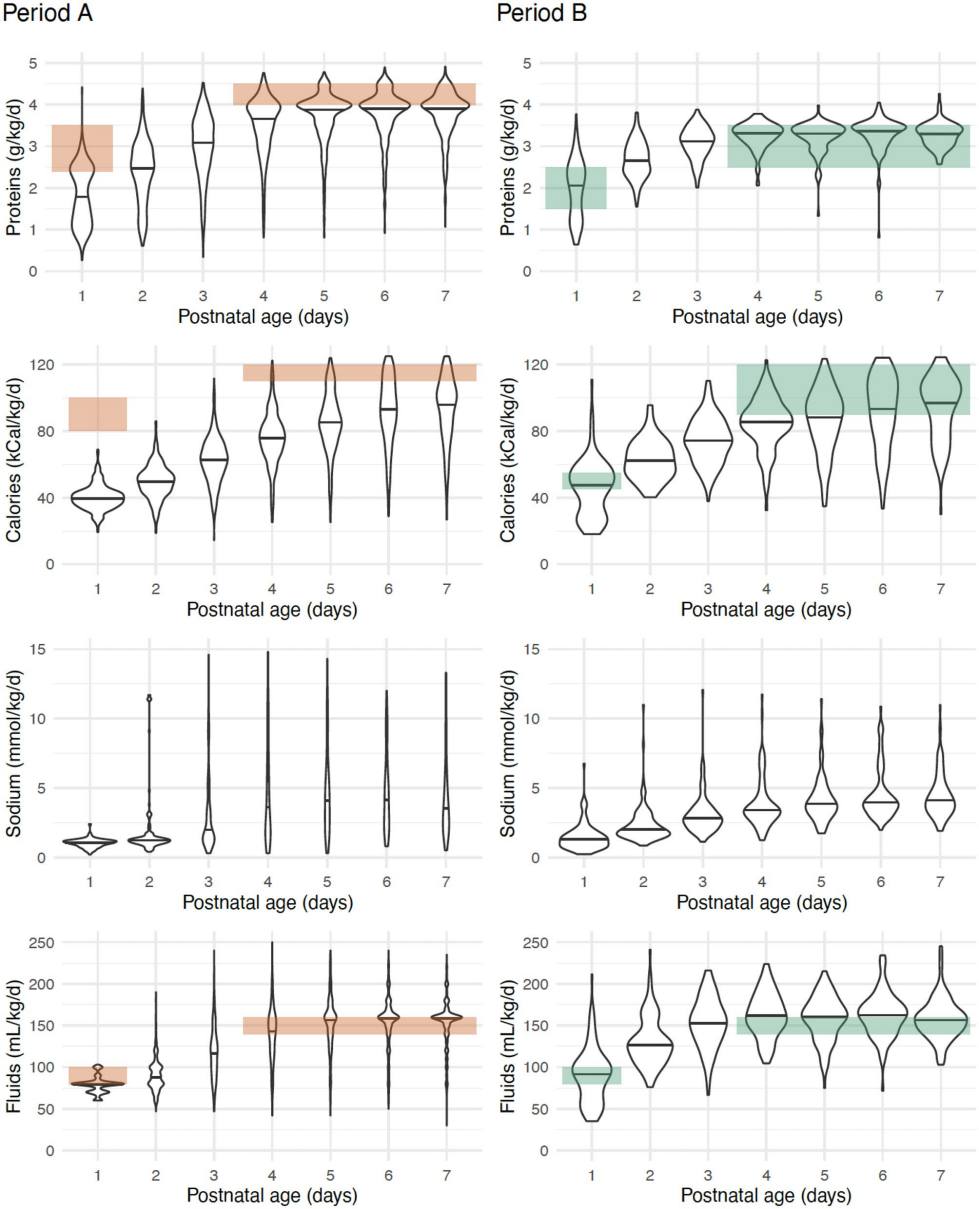
Nutritional intakes in the first week according to postnatal age in periods **A** and **B**. The shaded areas highlight ESPGHAN 2005 (light red) and 2018 (light green) guidelines at Day 1 and Days 5–7

Significant differences in macronutrient intake were observed between the two periods (Fig. 2). The median protein intake was 3.86 g/kg/d and 3.18 g/kg/day between D5 and D7 in periods A and B, respectively (*p* < 0.001), and the caloric intake was 80.6 kcal/kg/day and 91.1 kcal/kg/day between D5 and D7 in periods A and B, respectively (*p* = 0.005). Notably, the 2005 European Society ESPGHAN recommended 4–4.5 g/kg/day protein intake, which was revised in 2018 so as not to exceed 3.5 g protein/kg/day.

Table 3 presents the number of neonates who met protein and caloric intake recommendations over time according to the ESPGHAN 2005 and 2018 guideline recommendations. Almost 44% of protein intakes were in the range of the 2005 recommendations from D5 to D7 in period A, whereas more than 40% to 50% fell below the 2005 recommendations. In contrast, almost all caloric intakes fell below the 2005 recommendations in the same period. There was a higher proportion of the “high” caloric intake from D5 to D7 in Period A than Period B. However, this trend was not observed for caloric intake on day 1. Besides, there may have been an anticipation of 2018 guidelines for protein starting dose on day 1, but this trend was not observed for protein intake on days 5 to 7.

**Table 3:** Nutritional intake during periods A and B and compliance to the guidelines

	D1			D5-D7		
	Period A	Period B	p.value	Period A	Period B	p.value
	*N* = 121	*N* = 97		*N* = 121	*N* = 97	
Protein intake	1.84 [1.20;2.43]	2.00 [1.23;2.45]	0.282	3.98 [3.71;4.14]	3.32 [3.13;3.44]	<0.001
Calories intake	38.2 [34.4;42.9]	46.0 [27.5;53.9]	0.006	83.1 [70.1;93.0]	97.4 [82.4;107]	<0.001
Protein compliance:						
High	0 (0%)	20 (21.1%)		2 (1.65%)	15 (15.5%)	
Adequate	34 (27.6%)	42 (44.2%)		53 (43.8%)	80 (82.5%)	
Low	89 (72.4%)	33 (34.7%)		66 (54.5%)	2 (2.06%)	
Calories compliance:						
High	0 (0%)	23 (24.2%)		0 (0%)	1 (1.03%)	
Adequate	0 (0%)	27 (28.4%)		2 (1.65%)	62 (63.9%)	
Low	123 (100%)	45 (47.4%)		119 (98.3%)	34 (35.1%)	

D1: first day of life; D5–D7: median between day 5 and day 7 of life

**Fig. 3:**
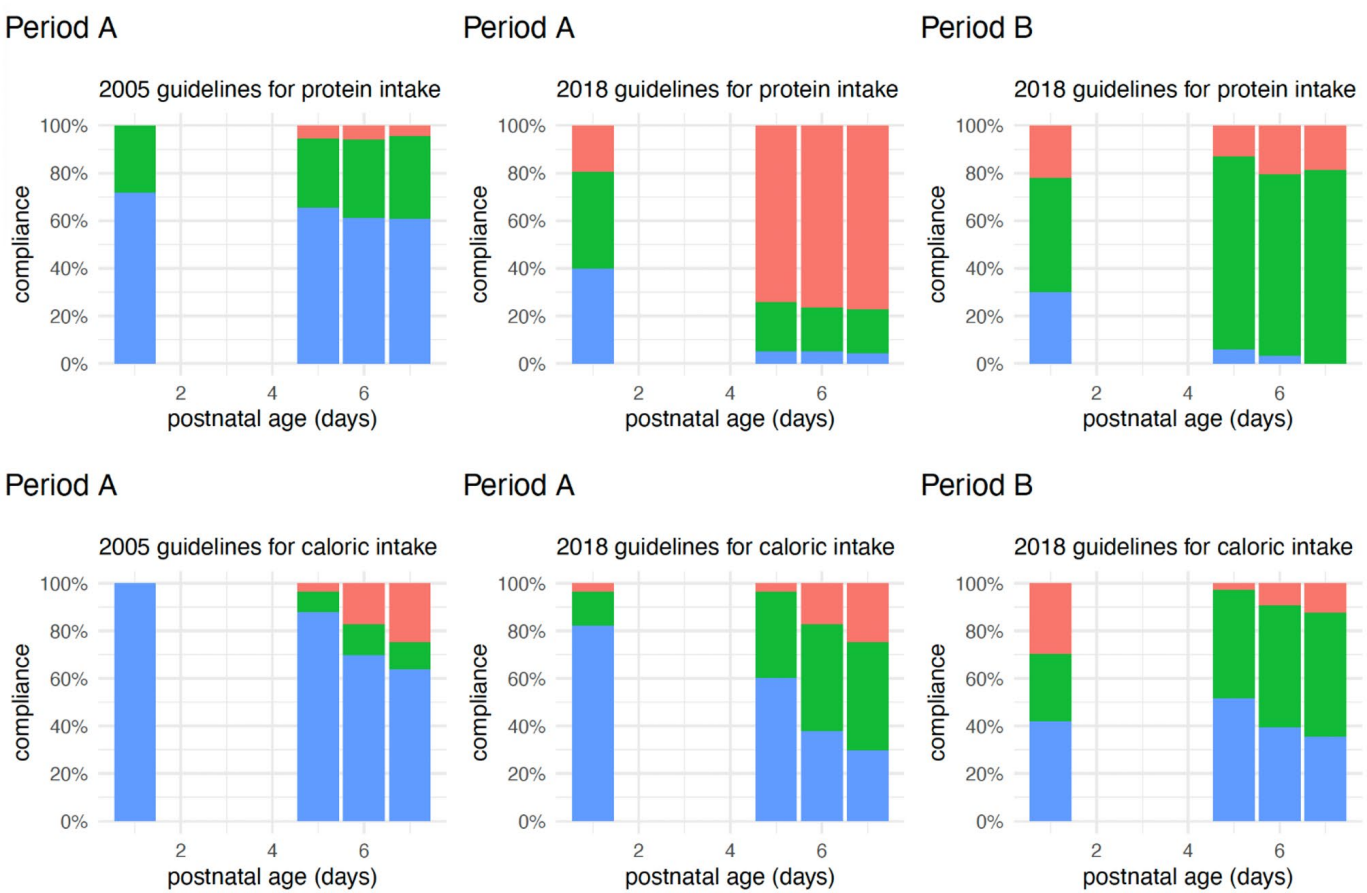
Compliance to ESPGHAN 2005 and 2018 recommendations during periods **A** and **B**. Protein (upper panels) and caloric (lower panels) intakes were represented over time according to postnatal age during the first week. Colors indicate whether the intakes fell below the recommended minimum intake (blue), above the recommended maximum intake (red), or within the appropriate range (green considered adequate)

A comparison between the left and right panels of Fig. 3 illustrates the increased compliance with guidelines over time. During period B, the majority (44.2% at D1 and 82.5% at D5–D7) of protein intakes were in the range of the 2018 recommendations, and more than 50% of caloric intakes were in the range of the 2018 recommendations at D5–D7. Compliance to guidelines is higher for the target dose, on days 5 to 7, than for the initial dose, on day 1. Yet the target dose was not always reached.

### Power analysis and minimal detectable effect

For compliance to guidelines in protein intake during the first two days of life (27% in Period A vs. 42% in Period B), the MDE was 16.95% points, meaning that a change from 27% to at least 43.95% could be detected with sufficient power. Given that the observed compliance was 42%, the study was at the threshold of detectability for this specific comparison.

For compliance to guidelines in protein intake between days 5 and 7 (44% in Period A vs. 82.5% in Period B), the MDE was 18.95% points, indicating that our study had sufficient power to detect an increase from 44% to at least 62.95%. Since the observed compliance was 80%, the study was adequately powered to detect this difference.

These results confirm that sufficient power was sufficient to detect the observed effect sizes for the primary compliance outcome. However, for smaller differences, particularly in the first day of life, the study was at the limit of detectability, suggesting that subtle improvements might not have been statistically distinguishable given the sample size.

### Assessment of postnatal growth and comorbidities

The matching weight (MW) method was the most effective at reducing the imbalance in baseline characteristics, as measured by the standardized mean differences (Appendix 3). After weighting, 97 (49 and 48) newborns were included in the noncompliance and compliance groups, respectively. The two groups were balanced (SMD < 0.1) for all variables describing the preintervention characteristics.

The results concerning comorbidities and postnatal growth at 36 weeks are presented in Table 4. A total of 23/48 (41%) patients in the compliance intake group had an EUGR at 36 weeks, whereas 33/49 (59%) patients in the noncompliance intake group had an EUGR after propensity score adjustment (*p* = 0.026). Regarding comorbidities, we identified a significant difference in the incidence of PDA with medical treatment and with surgical treatment between the two groups (34% in the compliance group vs 66% in the noncompliance group and 21% vs 79%, respectively).

**Table 4:** Comparison of postnatal growth and comorbidities according to compliance to guidelines in all population

	All population					Weighted population				
	Compliance *N* = 53	Noncompliance *N* = 165	*p* value	SMD	Missing	Compliance *N* = 49	Noncompliance *N* = 48	*p* value	SMD	Missing
**Postnatal growth**										
EUGR at 36 weeks			0.152	0.283	18			0.026	0.413	17
Yes	25 (61.0)	103 (74.1)				23 (41%)	33 (59%)			
No	16 (39.0)	36 (25.9)				15 (63%)	9 (37%)			
Weight delta Z-score at 36 weeks	−1.16 [−1.61 -0.76]	−1.28 [−1.67 -0.97]	0.168	0.284	18	−1.16 (−1.63 -0.79)	−1.28 (−1.62 -1.03)	0.2	0,289	17
**Comorbidities**										
BPD			0.483	0.152	16			0.7	0,077	11
Yes	27 (62.8)	88 (55.3)				26 (48%)	29 (52%)			
No	16 (37.2)	71 (44.7)				13 (44%)	17 (56%)			
NEC			0.206	0.231	6			0.13	0,267	4
Yes	12 (24.5)	25 (15.3)				12 (62%)	7 (38%)			
No	37 (75.5)	138 (84.7)				34 (46%)	40 (54%)			
PDA treated			0.009	0.469	1			0.016	0,447	0
Yes	11 (21.2)	70 (42.4)				11 (34%)	20 (66%)			
No	41 (78.8)	95 (57.6)				38 (58%)	28 (42%)			
PDA with surgical treatment			0.046	0.409	1			0.041	0,402	0
Yes	2 (3.8)	26 (15.8)				2 (21%)	8 (79%)			
No	50 (96.2)	139 (84.2)				47 (54%)	40 (46%)			
Neuroimaging lesions			0.333	0.239	27			0.15	0,302	12
Yes	2 (4.5)	16 (10.9)				2 (25%)	6 (75%)			
No	42 (95.5)	131 (89.1)				39 (50%)	38 (50%)			
ROP4			1.000	0.110	0			0.3	0,078	0
Yes	0 (0.0)	1 (0.6)				0 (0%)	0 (0%)			
No	53 (100.0)	164 (99.4)				49 (100%)	48 (100%)			
BSI			0.468	0.203	0.0			0.6	0,147	0
No	44 (83.0)	124 (75.2)				41 (52%)	37 (48%)			
1	8 (15.1)	38 (23.0)				8 (43%)	10 (57%)			
2	1 (1.9)	3 (1.8)				0 (37%)	1 (63%)			
CLABSI			0.397	0.215	0.0			0.5	0,107	0
Yes	1 (1.9)	10 (6.1)				1 (35%)	2 (65%)			
No	52 (98.1)	155 (93.9)				48 (51%)	46 (49%)			
Severe comorbidities			1.000	0.021	27			0.6	0,083	12
Yes	28 (63.6)	95 (64.6)				27 (47%)	31 (53%)			
No	16 (36.4)	52 (35.4)				13 (51%)	13 (49%)			
Death before discharge			1.000	0.018	0.0			0.8	0,056	0
Yes	8 (15.1)	26 (15.8)				8 (54%)	7 (46%)			
No	45 (84.9)	139 (84.2)				41 (50%)	41 (50%)			
Death or comorbidities			0.924	0.043	7			0.8	0,048	3
Yes	35 (68.6)	113 (70.6)				34 (50%)	34 (50%)			
No	16 (31.4)	47 (29.4)				13 (53%)	12 (47%)			

BSI: bacteremia, CLASBI: central line-associated bacteremia, PDA: Patent ductus arteriosus, BPD: bronchopulmonary dysplasia, NEC: Necrotizing enterocolitis, EUGR: extrauterine growth restriction, ROP: retinopathy of prematurity

There was a significant correlation between noncompliance caloric and/or protein intake and the weight delta Z-score at 36 weeks (beta − 0.13; 95% CI −0.26 - 0.00; *p* = 0.045) (Table 5). Although EUGR at 36 weeks (weight delta Z-score < −1) was observed more frequently in neonates exposed to noncompliance intake, this association did not reach statistical significance after adjusting for GA, BW and weighting by MW (OR 2.6; 95% CI 0.95–7.55; *p* = 0.068) (Table 5). There was no significant association between noncompliance intake and the risk of “death” or “severe comorbidity” after adjustment for or weighting by the MW.

**Table 5:** Association between noncompliance intake on days 5 to 7 and growth at 36 weeks of postmenstrual age. Weighted models use the MW method based on the propensity score. Weighted and adjusted models are additionally adjusted for gestational age and birth weight. CI: confidence interval; OR: odds ratio

	Weight Delta Z-Score At 36 Weeks Of Pma		Eugr At 36 Weeks Of Pma	
	BETA (95% CI)	*p* Value	OR (95% CI)	*p* Value
Unweighted models	−0.15 (−0.33–0.44)	0.118	1.8 (0.87–3.8)	0.106
Weighted models	−0.14 (−0.28–0.00)	0.053	2.47 (0.93–6.87)	0.074
Weighted and adjusted models	−0.13 (−0.26–0.00)	0.045	2.6 (0.95–7.55)	0.068

All population represents the population before weighting by the MW transform of the propensity score. Weighted population represents the population after weighting by the MW transform of the propensity score.

The weighting process allows for a more balanced comparison of outcomes between the two populations (Compliance vs. Noncompliance intakes) by reducing confounding biases related to patients’ initial conditions.

## Discussion

### Main results

In our retrospective monocentric study, we found that compliance to the ESPGHAN guideline recommendations for parenteral nutrition increased from 43.3% to 82.5% and to 1.65% to 63.9% regarding protein and caloric intakes at day 5 to 7 respectively after the implementation of an electronic medical health record. Noncompliance protein or caloric intake between D5 and D7 was significantly associated with reduced postnatal growth velocity, as assessed by the delta Z-score of weight between birth and 36 weeks. However, noncompliance intake was not associated with an increased risk of EUGR, death or major comorbidity before discharge in our population.

### Clinical relevance

During period A, less than 50% of protein intake and almost all caloric intakes were below the 2005 recommendations. In contrast, during period B (between D5 and D7), most protein intakes and more than 50% of caloric intakes fell within the 2018 ESPGHAN recommendations. Notably, while the number of patients receiving recommended energy provisions increased between 2005 and 2018, nearly 50% of caloric intake still fell short of the 2018 recommendations. This increase in recommendation compliance at D1 and D5–D7 was allowed by the simultaneous implementation of the Metavision® prescription software in our NICU. Some authors have already demonstrated that the use of computerized ordering systems can increase the quality of prescriptions closer to goals [24] as well as the safety of neonatal prescriptions [25] and improve postnatal growth in weight [26]. Lehmann et al. reported an 89% reduction in the error rate after the development of an online PN calculator, especially for calculation errors [27]. However, a study has shown that the rate of prescription errors may increase with time after the implementation of a computerized physician order entry, and repeated revision of the system could be useful [28]. In our experience, the lower compliance in Period A, despite protocol availability and staff training, likely stemmed from the mathematical complexity of manually adjusting standardized solutions to meet ambitious targets. Without real-time decision support, clinicians often adopt more conservative prescribing patterns. The MetaVision software bridged this gap by reducing cognitive load, providing immediate feedback on nutritional deficits, and automating complex calculations, thus facilitating the bedside application of theoretical guidelines.

Our results also revealed that noncompliance protein and/or caloric intake between D5 and D7 was correlated with a lower weight delta Z-score at 36 weeks. This finding is consistent with literature. Wang et al. showed that poor weight gain in very-low-birth-weight infants was related to insufficient parenteral macronutrient and energy intake [29]. Several studies have shown an improvement in growth with a higher intake of protein and calories [30, 31]. Furthermore, an observational study showed that high caloric intake can be a protective factor against EUGR [32], and a randomized trial reported a reduction in the incidence of EUGR with high fat intake, which is the main source of caloric intake [33]. Iacobelli et al. identified cumulative amino acid intake as a variable associated with the delta Z-score for weight at 36 weeks, but the population of the study included moderately preterm infants [34].

### Strengths and limitations

This study has several limitations. This was a retrospective monocentric study with a small sample size, which may have led to a lack of power. The absence of a significant difference in EUGR after weighting for initial conditions and adjusting for birth weight and gestational age does not rule out the effect of noncompliance caloric and/or protein intake on postnatal growth. This is because the weighting procedure reduced the effective sample size, thereby decreasing the statistical power of the analysis.

Combining PN and EN intakes to analyze the ESPGHAN’s 2018 PN recommendations is a compromise because EN is introduced as soon as possible, so total parenteral nutrition is delivered for a short time. However, there is a lack of current guidelines for combined enteral and parenteral nutrition.

In our study, we showed an improvement in compliance with recommendations during period B, but it should be noted that recommendations changed between the two periods, with lower recommended amino acid intakes and a larger range of caloric intakes. It seems to be easier to follow the ESPGHAN 2018 recommendations, so we cannot exclude that this may have had an impact on improved compliance.

Since day 1 is defined based on intake from 4pm to 4pm, our data may overestimate intakes by partially including those from the second day. This limitation could contribute to the lower compliance observed on day 1 compared to days 5 to 7.

One of the strengths of this study is the use of a propensity score, which allowed us to minimize the effect of known confounders that may have influenced treatment assignments. The use of standardized mean differences, which allowed us to check the balance of the two groups after weighting the propensity score, is recommended rather than the use of *p* values [35]. A comparative analysis after weighting by propensity score made the two groups comparable in terms of their initial characteristics.

Assessing postnatal growth using the weight delta Z-score is more strongly associated with neurodevelopmental outcome at 24 months than using a cross-sectional approach [19, 36]. We could have used a long-term outcome, but using a short-term outcome allows us to limit confounding factors.

Finally, our study did not specifically evaluate the technical constraints or potential drawbacks of the MetaVision software itself. In addition, compliance with nutritional recommendations was intentionally assessed in a descriptive manner and was not weighted according to clinical events requiring fluid restriction, such as patent ductus arteriosus, which may have constrained total fluid volume and consequently limited caloric intake in some patients.

### Perspectives

Our study primarily evaluates compliance with nutritional support guidelines rather than true adherence, which would require assessing the intentional engagement of healthcare providers in following and sustaining the recommendations over time. Future research could explore adherence more comprehensively by integrating qualitative assessments of behavioral commitment and implementation strategies.

The next step would be to assess these results in a larger population in a multicenter study or over a longer observation period to determine the effect of variations in potential local practices.

Assessment of postnatal growth using length could be interesting because this measure has been shown to be associated with 2-year neurodevelopmental outcomes [37]. However, length is a more variable measure because of interindividual variation between professionals.

In any case, the results of this study may encourage the use of computerized prescription software to enhance compliance with guideline recommendations.

## Conclusion

The deployment of individualized prescription software has increased compliance with nutritional recommendations in an extremely low birth weight preterm infant population. In our population, caloric and protein intakes in line with the 2018 ESPGHAN guidelines for preterm newborns were associated with a significant change in postnatal growth at 36 weeks. The effects of implementing prescription software on postnatal growth remain to be evaluated in a larger cohort.

## Electronic supplementary material

Below is the link to the electronic supplementary material.

Supplementary material 1

## Data Availability

The datasets used and/or analyzed during the current study are available from the corresponding author upon reasonable request.

## Abbreviations

BL: Birth length
BSI: Bacteremia
BPD: Bronchopulmonary dysplasia
BW: Birth weight
CLASBI: Central line-associated bacteremia
EUGR: Extrauterine growth restriction
ESPGHAN: European Society for Pediatric Gastroenterology Hepatology and Nutrition
GA: Gestational age
HC: Head circumference
MW: Matching weight
NEC: Necrotizing enterocolitis
NICU: Neonatal intensive care unit
PDA: Patent ductus arteriosus
PN: Parenteral nutrition
PROM: Prolonged rupture of membranes
SMD: Standardized median deviation
