# Supplementary material for: Assessment of compliance to the guidelines on nutritional support in a neonatal intensive care unit: association with an electronic health Records implementation
Source: BMC Med Inform Decis Mak. 2026 May 14;26:250. doi: 10.1186/s12911-026-03533-x (PMC13348983; doi:10.1186/s12911-026-03533-x)

**Appendix 1.** Composition of parental nutritional solutions

| **For 100 mL** | **PEDIAVEN APHP NN1 ®** | **PEDIAVEN APHP NN 2 ®** | **Numetah G13%E 3 CP ®** | **Numetah G13%E 2 CP ®** | **Numetah G16%E 3CP®** | **Numetah G16%E 2CP ®** | **SNPN1 ®** |
| --- | --- | --- | --- | --- | --- | --- | --- |
| **Producer** | Fresenius Kabi | Fresenius Kabi | Baxter | Baxter | Baxter | Baxter |  |
| **Glucose (g)** | 10 | 10 | 13,3 | 16,7 | 15,5 | 20,6 | 15 |
| **Carbohydrates (g)** | 1,5 | 1,7 | 3,1 | 3,9 | 2,6 | 3,5 | 6 |
| **Lipids (g)** | 0 | 0 | 2,5 | 0 | 3,1 | 0 | 0 |
| **Calories (kcal)** | 46 | 79 | 91 | 82 | 93 | 96 | 84 |
| **Osmolarity (Osm)** | 715 | 790 | 1150 | 1410 | 1230 | 1585 | 1364,5 |
| **Sodium (mmol)** | 0,45 | 2 | 2,2 | 2,7 | 2,4 | 3,1 | 3 |
| **Potassium (mmol)** | 0 | 1,7 | 2,1 | 2,6 | 2,28 | 3 | 0 |
| **Calcium (mmol)** | 0,94 | 0,76 | 1,3 | 1,6 | 0,62 | 0,82 | 0 |
| **Magnesium (mmol)** | 0,21 | 0,16 | 0,16 | 0,2 | 0,32 | 0,41 | 0,3 |
| **Chlorure (mmol)** | 0,5 | 2,6 | 3,1 | 3,9 | 2,8 | 3,7 |  |
| **Phosphore (mmol)** | 0 | 0,91 | 1,3 | 1,3 | 0,88 | 0,85 | 2 |
| **Micronutrient** | Yes | Yes | No | No | No | No | Yes |
| **Vitamins** | No | No | No | No | No | No | No |

| **Amino Acid composition** | | | | | |
| --- | --- | --- | --- | --- | --- |
| **Active substance** | **PEDIAVEN APHP NN1 ®** | **PEDIAVEN APHP NN 2 ®** | **Numetah G13%E ®** | **Numetah G16%E ®** | **SNPN** |
| Alanine | 0.36g | 0.41g | 0.75 g | 1,03 g | 0.7 g |
| Arginine | 0.24g | 0.27g | 0.78 g | 1,08 g | 0.7 g |
| Aspartic acid | 0.24g | 0.27g | 0,56 g | 0,77 g | 0.5 g |
| Cystéine | 0.06g | 0.07g | 0,18 g | 0,24 g | 0.16 g |
| Glutamic acid | 0.41g | 0.46g | 0.93 g | 1,29 g | 0.84 g |
| Glycine | 0.12g | 0.14g | 0,37 g | 0,51 g | 0.34 g |
| Histidine | 0.12g | 0.14g | 0,35 g | 0,49 g | 0.32 g |
| Isoleucine | 0.18g | 0.2g | 0,62 g | 0,86 g | 0.56 g |
| Leucine | 0.4g | 0.46g | 0.93 g | 1,29 g | 0.84 g |
| Lysine | 0.32g | 0.36g | 1,03 g | 1,42 g | 0.92 g |
| Methionine | 0.07g | 0.08g | 0,22 g | 0,31 g | 0.2 g |
| Ornithine | / | / | 0,23 g | 0,32 g | 0.27 g |
| Phenylalanine | 0.16g | 0.18g | 0,39 g | 0,54 g | 0.35 g |
| Proline | 0.32g | 0.36g | 0,28 g | 0,39 g | 0.25 g |
| Serine | 0.22g | 0.25 | 0,37 g | 0,51 g | 0.34 g |
| Taurine | 0.02g | 0.02g | 0,06 g | 0,08 g | 0.05 g |
| Threonine | 0.21g | 0.23g | 0,35 g | 0,48 g | 0.31 g |
| Tryptophan | 0.08g | 0.09g | 0,19 g | 0,26 g | 0.17 g |
| Tyrosine | 0.03g | 0.03g | 0,07 g | 0,10 g | 0.04 g |
| Valine | 0.21g | 0.23g | 0,71 g | 0,98 g | 0.64 g |

| Producer | **Clinoléic ®**  **Baxter** | **SMOF Lipids ®**  **Fresenius Kabi** |
| --- | --- | --- |
| **TCL soja** | 20% | 30% |
| **TCL olive** | 80% | 25% |
| **TCM (coconut/palme)** |  | 30% |
| **TG à w3 (fish)** |  | 15% |

**Appendix 2**. Screenshots of the nutritional information provided by the Metavision prescription software


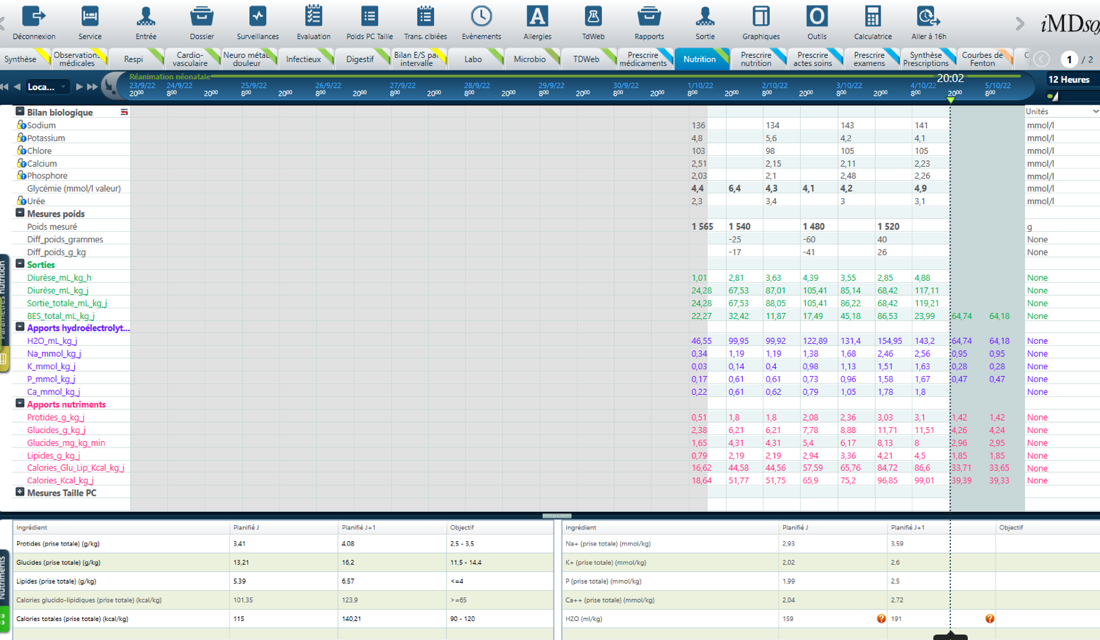


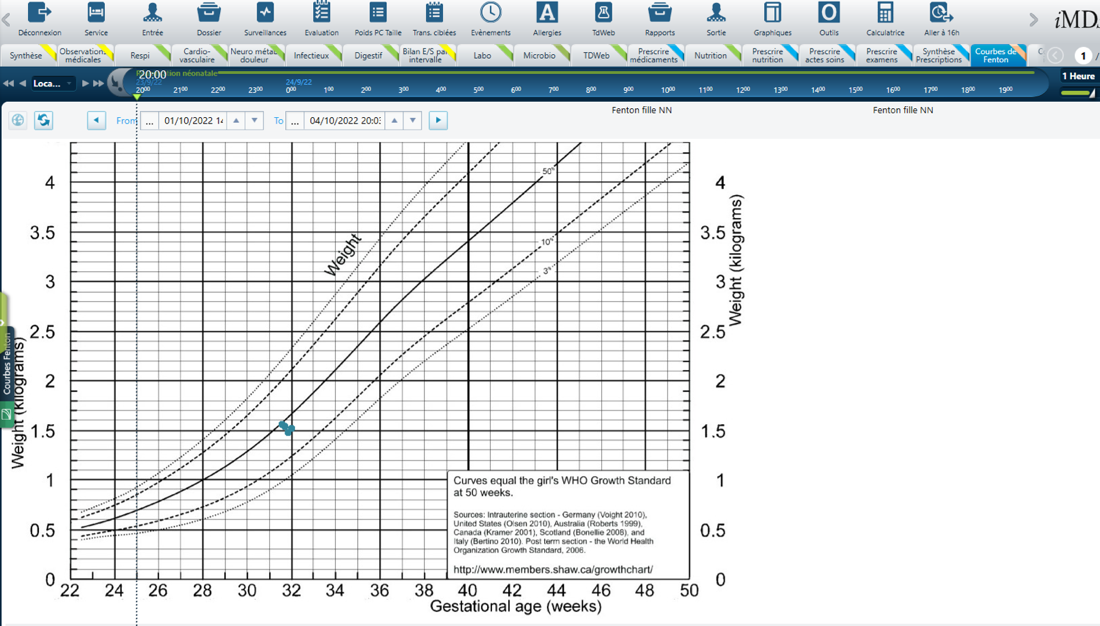


**Appendix 3.** The quality of weighting and matching was verified by estimation of SMDs, revealing the absence of residual imbalance

The propensity score was estimated using a logistic regression model with caloric and/or protein intake as the dependent variable with respect to the following characteristics: gestational age, sex, BW Z score, antenatal corticosteroid therapy, PROM over 24 hours, preeclampsia, multiparity, chorioamniotitis, mode of birth, 1- and 5-minute APGAR, intubation, and surfactant instillation.


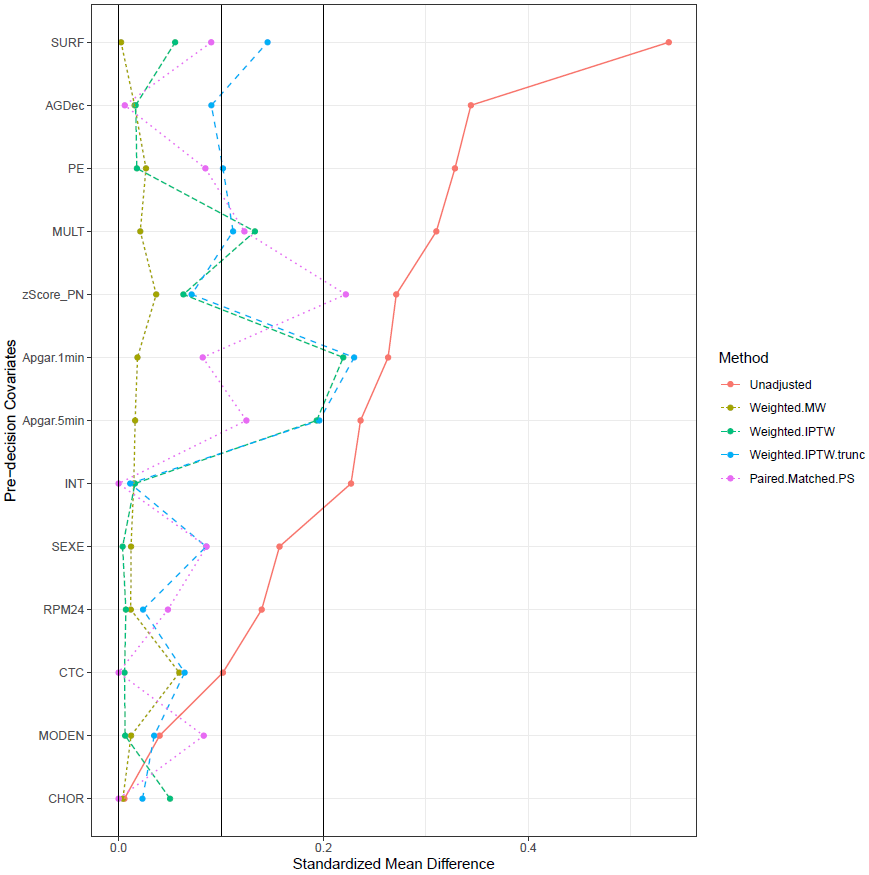

Supplement: Supplementary file 1 — Supplementary material 1 [file 12911_2026_3533_MOESM1_ESM.docx]
